# Editorial: The role of radiotherapy in reshaping tumor immune microenvironment

**DOI:** 10.3389/fimmu.2023.1340844

**Published:** 2023-12-05

**Authors:** Xiaoyu Hu, Mengyu Zhao, Jinbo Yue

**Affiliations:** Department of Radiation Oncology, Shandong Cancer Hospital and Institute, Shandong First Medical University and Shandong Academy of Medical Sciences, Jinan, Shandong, China

**Keywords:** radiotherapy, immunotherapy, immune microenvironment, autophagy, colorectal cancer immunotherapy, represented by immune checkpoint inhibitors (ICIs)

Immunotherapy, represented by immune checkpoint inhibitors (ICIs), has improved outcomes in several types of cancers. However, many patients still do not respond (1). The anti-tumor response mediated by ICIs depends on the infiltration of cytotoxic T lymphocytes (CTLs) that can recognize and kill tumor cells (2). Tumors can be classified into three primary immune phenotypes based on the spatial distribution of CTLs within the tumor microenvironment (TME): these are the immunoinflammatory phenotype, the immunoexclusionary phenotype, and the immune-desert phenotype (1). Immunoinflammatory tumors, a.k.a “hot tumors”, are characterized by substantial T cell infiltration, amplified IFNγ signaling, and a high tumor mutation burden (TMB). These tumors typically show increased sensitivity to ICIs (3). Conversely, immunoexclusionary and immune-desert tumors, which have less immune activity, are often termed “cold tumors”.

Radiotherapy (RT) can not only directly destroy tumors but also induce a systemic immune response. Radiation induces immunogenic death in tumor cells, activates anti-tumor immune responses, and produces an effect similar to an “*in situ* vaccine” (4). Moreover, RT has a multifaceted regulatory effect on immune cells and tumor stroma cells. It can also influence the TME by inducing production of various cytokines, ultimately transforming cold tumors into hot and enhancing the efficacy of immunotherapy (5). RT can reshape the immune TME from multiple perspectives, although its specific mechanisms have not been fully elucidated.

In this Research Topic, multiple studies investigate the role of RT in remodeling the immune TME. Gao et al. have identified three autophagy-related genes—SHC1, NAPSA, and AURKA—associated with RT and the prognosis of lung adenocarcinoma. NAPSA and AURKA were also closely associated with T_H_2 cell infiltration. T_H_2 cells promote tumor activity by facilitating angiogenesis and inhibiting the cell-mediated immune response that destroys tumor cells. Accumulating evidence suggests that autophagic activity can regulate immune cell infiltration by modulating both the innate and adaptive immune systems. Therefore, it is possible that autophagy-related genes may inhibit antitumor immunity by promoting T_H_2 cell infiltration following RT. Hence, targeting autophagy-related genes could represent a novel immunomodulatory strategy to enhance the effectiveness of RT in patients with lung adenocarcinoma.

Shi et al. provide a summary of radioimmunotherapy in the treatment of colorectal cancer, discussing its current status and future prospects. Patients with microsatellite instability (MSI) comprise less than 5% of rectal cancer cases, while the majority of colorectal cancers are of the microsatellite stable (MSS) type. These MSS patients derive little-to-no benefit from immunotherapy alone. Due to the synergistic effect between RT and immunotherapy, RT can release tumor antigens, reshape the immune microenvironment, and increase the anti-tumor immune response, thereby producing a synergistic therapeutic effect. Numerous prospective phase II studies suggest that the combination of RT with immunotherapy, used for pMMR/MSS locally advanced rectal cancer, has achieved a higher rate of complete pathological response (pCR) (6).

Chi et al. show that hRT’s regulation of the immune microenvironment depends on the dosage and fractionation pattern of the RT. RT can potentiate antigen presentation, leading to the upregulation of pro-inflammatory cytokine levels and cytotoxic T cell-mediated immunogenic cell death. However, RT can also induce immunosuppressive responses, characterized by the augmented recruitment and infiltration of regulatory T cells, myeloid-derived suppressor cells (MDSCs), and molecules associated with immune checkpoints. Combining RT with immunotherapy may shift this balance, tipping it towards immune activation. This combination could potentially overcome resistance to ICIs and other forms of immunotherapy. Strategies to enhance the efficacy of such combinations warrant further exploration.

In recent years, numerous *in vivo* studies have revealed the synergistic tumoricidal effects of combining RT with PD-1/-L1 inhibitors. The dynamic nature of the PD-1/-L1 axis, however, has confounded efforts towards a consensus on the optimal timing or dose-fractionation strategy for doing so (6). Understanding the synergistic effects of combining RT with PD-1/PD-L1 blockade requires investigation into the dynamic changes in tumor immune status and circulating biomarkers induced by RT in relation to PD-1/PD-L1 expression. Yoon et al. demonstrate that, following RT, PD-L1 expression levels in tumor cells and intratumoral MDSCs, TAMs, and DCs are upregulated, mainly during the period of tumor regression. With tumor regrowth, PD-L1 levels in tumor cells were found to decrease to levels comparable to those at baseline.

Our understanding of how RT can reshape the immune TME across various levels has continued to grow, informed by a legion of molecular and animal studies as well as clinical trials. As research in this area progresses, it is anticipated that the confluence of RT and immunotherapy will yield increasingly potent therapeutic interactions.

## Author contributions

XH: Writing – original draft. MZ: Writing – original draft. JY: Writing – review & editing.
